# Applicant hierarchical fuzzy controller for concentration control of simulated moving bed

**DOI:** 10.1038/s41598-021-97134-5

**Published:** 2021-09-02

**Authors:** Chaofan Xie, Yang-jie Tang

**Affiliations:** 1 Fujian Polytechnic Normal University, Big Data and Artificial Intelligence of Fujian Polytechnic Normal University, Fuzhou, 350300 China; 2 Zhangzhou Normal University, School of Mathematics and Statistics, Zhangzhou, 350121 China

**Keywords:** Applied mathematics, Computational science, Chemical engineering

## Abstract

Simulated moving bed (SMB) is a kind of continuous process which can increase the efficiency of adsorbents in the adsorbent bed. It contains several sectors of flow rate, the switching time of valves and many other possible influencing variables, moreover, these parameters are highly sensitive, so it is very difficult to achieve precise prediction and control. Model predictive control and PID controller are often used in industrial system. Model predictive control needs a lot of accurate industry experience data, and PID controller depends on the selection of control parameters. Therefore, SMB needs an intelligent controller to bypass those complex mechanisms and parameter adjustment processes. This paper we propose the hierarchical fuzzy controller fuzzy controller which is applied to the SMB system to observe the final concentration. Compared with the PID and MPC controller, it is found that the hierarchical fuzzy controller can control good without knowing the system parameters too accurately.

## Introduction

In chromatographic separation technology, mobile phase circulates against “pressure drop”. Pressure drop can be caused by either gravity or packing resistance. The solid adsorbent moves from top to bottom due to its own pressure drop, so that the mobile phase can fully contact with the stationary phase. In this way, due to the different adsorption capacity of different components, components flow out at different outlet positions. But the bed will inevitably produce wear in the countercurrent and generate a lot of powder. If the powder flows into the pipe, the pipe will be blocked and the flow rate will drop. In this way, the Sorbex system developed by UOP company in the 1960s combines the advantages of previous beds and avoids their disadvantages, which is the early simulated moving bed^1–3^.

Because of its high production capacity, low water and solvent consumption, and is considered as the cleanest production technology, simulated mobile bed is widely used in chemical and biopharmaceutical engineering^4^. Due to the low efficiency of computational hardware, early researchers focused on the development true moving bed (TMB) of mathematical model^5,6^. Later, the cost of hardware is gradually become low, the research on SMB and its application increased^7–9^.

**Figure 1:**
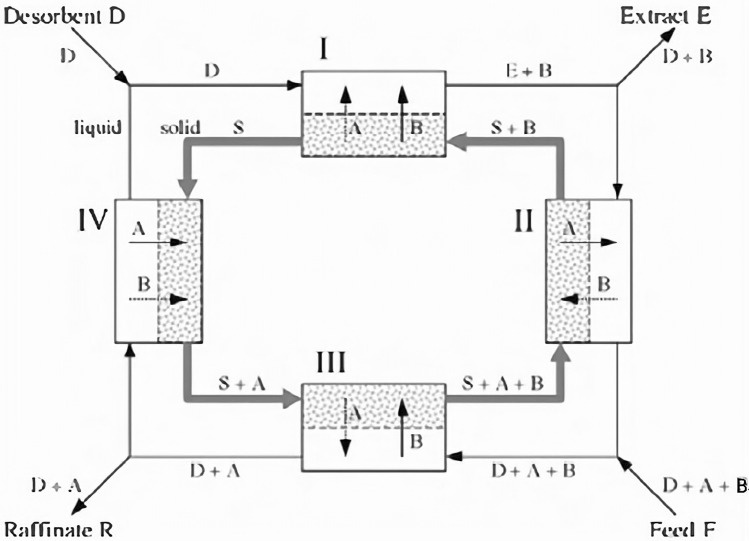
Simulated moving bed operation process.

As shown in Fig. 1, SMB unit is a system composed of a series of fixed bed towers, in which the loop pump flows around the solvent. In addition, one pump delivers fresh solvent to the system and the other pump delivers mixture to the system. There are two pumps between the solvent and feed position for extraction of separation and residues. The mobile phase and the stationary phase are formed by the periodic switching of the inlet and outlet flow, and the switching direction is consistent with that of the mobile phase. The mobile phase flows in the chromatographic column and forms a circulation through the interface connection. The switching time of each period is constant, its operation mainly includes four regions, for example, feeds are switched from C4 and C5 to C5 and C6, the other entrances and exits also move to the next pipe string at the same time, therefore, a similar purpose will be formed to make the solid counter clockwise. Continuously changing the position of the entrance and exit, so the counterclockwise drift of solid adsorbent is formed, however the moving phase is continuously clockwise. The process of reaching the same solid as TMB and continuously converse contact with the flow.

## Literature reviews

The technology of model predictive control has been widely used in chemical process control. Therefore, the application of model predictive control in SMB control has also been discussed in^10–13^. However, due to the high sensitivity of the SMB system, these methods are inefficient, and a small change in parameters will result in a significant change in the performance index (purity, recovery efficiency, etc.), because the parameter changes require a long time of training to converge, and the parameter changes may not be a slow process. An optimization strategy based on the improved moving asymptote algorithm is proposed by Yonghui Yang etc.^14^. The research shows that the optimized controller based on the improved moving asymptote method can dynamically control and optimize the process of the simulated moving bed, which is conducive to the design and operation of the simulated moving bed. In the practical application of SMB, Lucas et al.^15^ predict the performance of the actual SMB unit by mathematical optimization method, and the separation purity of TIA and GCA reached 97%. In reference^16^, Wei et al. not only studied the separation purity, but also studied the separation efficiency, and proposed a two-step separation method of feeding, purification and recovery. Combined with crystallization operation, the purity was further improved to 97.8%, and the yield reached 95%. In^17^, Maruyama et al. proposed a new method called bypass simulation moving bed. It provides additional freedom and opportunities to improve productivity. All the application research is carried out for real substances, so it is not a common method.

Kloppenburg et al. used a simple P controller and used the difference of UV spectrum waveform as the adjustment of real-time online SMB control parameters^18^. Bijan et al. proposed the design and optimal control of single column chromatographic process. The real-time online monitoring system detects the UV signal value, sends the signal value back to the system state estimator, generates the chromatographic separation process, sends out the best control signal, and controls the single column chromatographic tube, so as to achieve the best chromate-graphic control^19^. Based on the concept of optimal control, Toumi et al. designed a nonlinear model predictive controller^20^. Suvarov et al. applied self-adjusting control, which can adjust the spatial position of adsorption and desorption wave by adjusting the flow rate and switching period, and then adjust the purity and productivity of extraction liquid and extraction flow. This kind of model predictive control technology has been widely used in chemical engineering control^21^. In the simulation study, Juweon Lee proposed a new method. The controller can estimate the current process state under the competitive Langmuir isotherm, and find the best operation condition of “switch by switch”^22^. Based on the state space model, zheng yan etc.^23^ find an effect method which is applied to SMB chromatographic separation process. It uses two space identification method including output error state space and subspace state space (N4SID) numerical algorithm to model the SMB separation process, and obtains the third-order and fourth-order state space production efficiency models of SMB separation process. Xie et al.^24^ proposed an adjusted fuzzy controller to control the concentration of separation at the outlet. Compared with the traditional fuzzy controller, its fluctuation range is small, but the improvement is not obvious from the perspective of cost performance, but it increases the computational overhead. Other research can be found in^25–36^.

The operation of SMB is difficult to achieve “steady state”, steady state of SMB refers to whether the average purity in a switching cycle is stable at a certain value. Here we study concentration control. The average concentration in a cycle cannot reach a steady state which is described in detail in Sect. 4.2. The goal of concentration control is to reduce the amplitude of oscillation. The main reasons are the combination of system components, mechanical control disturbances and adsorbents. Factors such as leveling and so on, the stability of separation of SMB system will be affected, and in serious cases, it will lead to bad production products, therefore, traditional SMB operations usually need to do some disposal to ensure the yield of products, such as reducing some production capacity and increasing solvent consumption. Also, in SMB operation process, it is needed to predict the accurate physical parameters (for example like linear adsorption relationship) of the substances, so that the theory can be applied to delimit the separable area and carry out the separation work. However, SMB cannot guarantee the smooth implementation of accurate separation when the analysis of physical parameters of separated substances is not accurate enough.

Due to these problems of SMB system, we will encounter more troubles when we use the traditional PID controller. Column packing non-uniformity can cause pressure/flow-rate oscillations. Regardless of the tuning, traditional PID controller may either respond too aggressively and hence aggravate the oscillation, or too slowly to provide the best performance. Because of the switch time, step asymmetry can be problematic for model-based controller. In Xylene purification process, such as the PAREX process, there are 24 columns, each is packed slightly different, and the 24 columns are arranged into two towers, two long transfer lines are used to connect the two towers, traditional model-based predictive controller often fail to deal with step asymmetry. Model predictive controller is always based on a fixed set of parameters, however, over time, resin degradation will kick in. Model predative controller needs to be tuned often to accommodate the decline of the stationary phase performance. Moreover, air bubbles can cause PID instruments to malfunction. For example, a lot of processes that uses Coriolis meters. Degassing of the liquid and cause the mass flow controller to generate unrealistic data. This problem is very hard to solve for traditional model-based controller or PID controller. Therefore, when the parameters of SMB system change, the fuzzy controller does not need to modify the corresponding parameters, which can maintain strong robustness. Moreover, SMB system needs a controller that does not pay so much attention to accurate control algorithm, but can make a good response to the system especially for the changes of switch time. For example, we don't need to know Newton's law of motion. Acrobats can still control the inverted bars accurately without falling down. Thirdly, in a particularly complex nonlinear system, the control of fuzzy controller can be smoother, so as to reduce the amplitude of oscillation. The fuzzy controller based on fuzzy mathematics has these advantages; this is the starting point of using fuzzy controller to study SMB.

**Figure 2:**
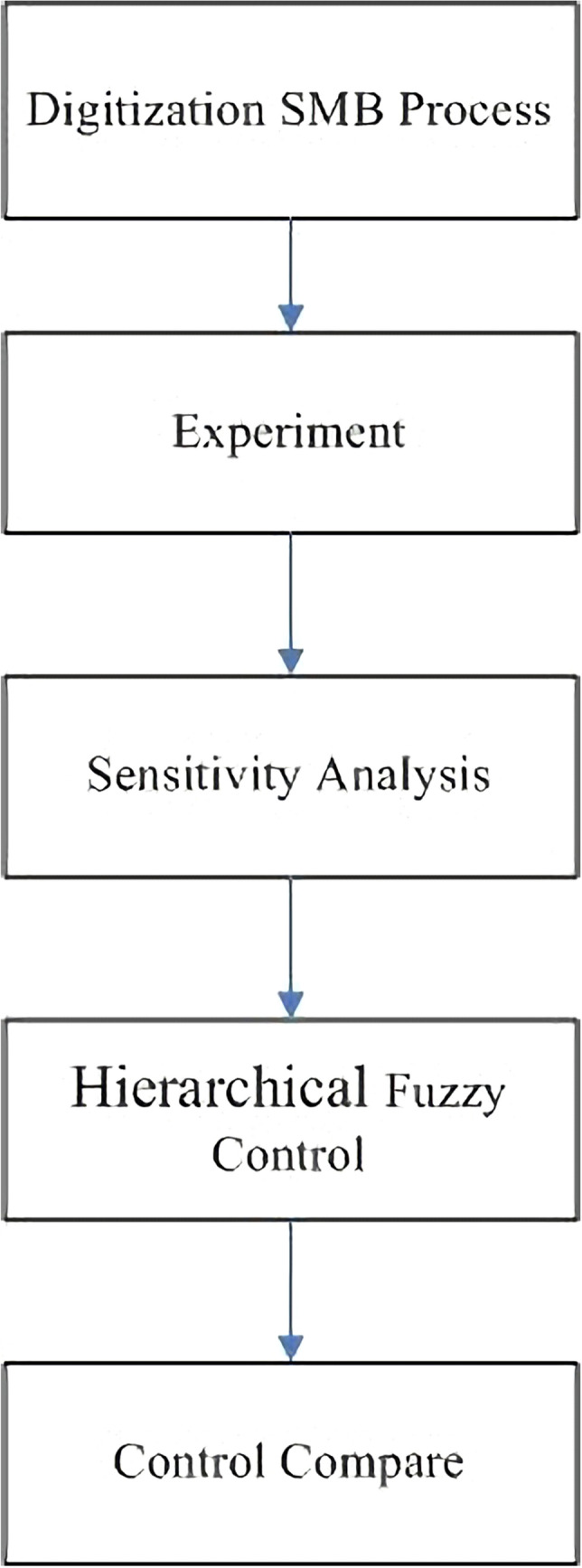
Research framework.

So, in this paper, a hierarchical fuzzy controller is proposed to control the separation concentration in the production process. Figure 2 shows the overall architecture of the paper. Firstly, through the digitized SMB process, we can observe whether the results obtained by using the finite element method are consistent. Then we can dynamically adjust the parameters in the process and observe the influence of the change of parameters on the final separation results. Third, it can control the parameter adjustment through the fuzzy rule multilevel hierarchy controller. Lastly, it compares with the PD and PID controller to observing whether the control effect is more advantageous.

## Related mathematical model and method

An overview of SMB dynamic model is given, which is mentioned in literature and the relevant symbols are described in Table 1. For TMB, the mass balance of bulk phase is:

1 $$ \frac{{\partial C_{i,j} }}{\partial t} = D_{i} \frac{{\partial^{2} C_{i,j} }}{{\partial x^{2} }} - v_{{_{j} }}^{*} \frac{{\partial C_{i,j} }}{\partial x} - \frac{1 - \varepsilon }{\varepsilon }k_{i} (q_{ij}^{*} - q_{ij} ) $$

2 $$ \frac{{\partial q_{i,j} }}{\partial t} = \frac{\partial }{\partial x}u_{s} q_{ij} + k_{i} (q_{ij}^{*} - q_{ij} ) $$

**Table 1:** Parameters of SMB system.

Parameter	Describe
*x* (cm)	Axial distance
*k* (g L^−1^)	Mass transfer constant
v* (cm min^−1^)	Effect velocity of column
*u*_*s*_ (cm min^−1^)	Solid velocity flow rate
*C* (g L^−1^)	Mobile phase concentration
*q* (g L^−1^)	Solid phase concentration
q* (g L^−1^)	Equilibrium concentration between solid phase and mobile phase
*Q* (cm^3^ min^−1^)	Flow rate
*t* (second)	Time
*D* (cm^2^ min^−1^)	Effective dispersion coefficient
*ε*	Bulk void fraction
*i*	Material index: A or B
*J*	Column number:1,2,3,4,5,6,7,8

So, TMB and SMB can be transformed to each other as follows:

3 $$ \frac{{\partial C_{i,j} }}{\partial t} = D_{i} \frac{{\partial^{2} C_{i,j} }}{{\partial x^{2} }} - v_{{_{j} }}^{*} \frac{{\partial C_{i,j} }}{\partial x} - \frac{1 - \varepsilon }{\varepsilon }k_{i} (q_{ij}^{*} - q_{ij} ) $$

4 $$ \frac{{\partial q_{i,j} }}{\partial t} = k_{i} (q_{ij}^{*} - q_{ij} ) $$

(4) substituted into (3), it gets as follows:

5 $$ \frac{{\partial C_{i,j} }}{\partial t} = D_{i} \frac{{\partial^{2} C_{i,j} }}{{\partial x^{2} }} - v_{{_{j} }}^{*} \frac{{\partial C_{i,j} }}{\partial x} - \frac{1 - \varepsilon }{\varepsilon }\frac{{\partial q_{i,j} }}{\partial t} $$

The adsorption equilibrium of the two enantiomers is expressed by linear isotherms.

6 $$ q(i,j) = H_{i} C(i,j) $$

The flow velocity in each region must satisfy the following conditions:

7 $$ \begin{gathered} Q_{I} { > }Q_{IV} ,Q_{I} { > }Q_{II} ,Q_{III} { > }Q_{IV} ,Q_{III} { > }Q_{IV} \hfill \\ Q_{I} { - }Q_{IV} { = }Q_{d} ,Q_{I} { - }Q_{II} { = }Q_{x} ,Q_{III} { - }Q_{IV} { = }Q_{r} ,Q_{III} { - }Q_{II} { = }Q_{f} \hfill \\ \end{gathered} $$

*Q*_*d*_, *Q*_*x*_, *Q*_*r*_ and *Q*_*f*_ are respectively the solvent, extract , raffinate and feed stream flow rates, also they can be selected as controllable variables.

### Using centered difference method to numerate PDEs

Since the mathematical model of SMB system are essentially diffusion equations, for this type, we use the centralized difference method because of its better stability and convergence, then using it to generate the iterative calculation formula of SMB discrete dynamic system.

Set $$t_{k} = t_{0} + ks$$, $$x_{l} = x_{0} + lh$$, $$F = \frac{1 - \varepsilon }{\varepsilon }$$

8 $$ \begin{aligned} \frac{{\partial^{2} C_{ij} }}{{\partial x^{2} }} = & \frac{{\left. {\frac{{\partial^{2} C_{ij} }}{{\partial x^{2} }}} \right|_{{t_{k} }} + \left. {\frac{{\partial^{2} C_{ij} }}{{\partial x^{2} }}} \right|_{{t_{k + 1} }} }}{{2}} \\ { = } & \frac{{C_{ij} (x_{l - 1} ,t_{{k{ + 1}}} ) - 2C_{ij} (x_{l} ,t_{{k{ + 1}}} ) + C_{ij} (x_{{l{ + }1}} ,t_{{k{ + 1}}} ){ + }C_{ij} (x_{l - 1} ,t_{k} ) - 2C_{ij} (x_{l} ,t_{k} ) + C_{ij} (x_{{l{ + }1}} ,t_{k} )}}{{{2}h^{2} }} \\ \end{aligned} $$

9 $$ \begin{gathered} \frac{{\partial C_{ij} }}{\partial x} = \frac{{\left. {\frac{{\partial C_{ij} }}{\partial x}} \right|_{{t_{k} }} + \left. {\frac{{\partial C_{ij} }}{\partial x}} \right|_{{t_{k + 1} }} }}{{2}} \hfill \\ { = }\frac{{C_{ij} (x_{l + 1} ,t_{{k{ + 1}}} ) - C_{ij} (x_{{l - {1}}} ,t_{{k{ + 1}}} ){ + }C_{ij} (x_{l + 1} ,t_{k} ) - C_{ij} (x_{{l - {1}}} ,t_{k} )}}{{{4}h}} \hfill \\ \end{gathered} $$

10 $$ \frac{{\partial C_{ij} }}{\partial t} = \frac{{C_{ij} (x_{l} ,t_{{k{ + }1}} ) - C_{ij} (x_{l} ,t_{k} )}}{s} $$

11 $$ \begin{aligned} & \frac{{\partial q_{ij} }}{\partial t}(x_{l} ,t_{k} ) = H_{i} \frac{{\partial C_{ij} }}{\partial t}(x_{l} ,t_{k} ) \\ & \quad \quad \quad i = 1, \ldots M,j = 1, \ldots N \\ \end{aligned} $$

*M, N* represent respectively number of material and zones, here, *M* = *2, N* = *8*, $t$ is time, $x$ is coordinate of the column, $s$ is time step, $h$ is space step. Substituted into SMB system, $C_{ij} (x_{l} ,t_{k} )$ denote as $C_{ij} (l,k)$ we can get:

12 $$ \begin{aligned} & \left( {1 + FH_{i} { + }\frac{Ds}{{h^{2} }}} \right)C_{ij} (l,k + 1) - \left( {\frac{vs}{{4h}} + \frac{Ds}{{2h^{2} }}} \right)C_{ij} (l - 1,k + 1) + \left( {\frac{vs}{{4h}} - \frac{Ds}{{2h^{2} }}} \right)C_{ij} (l - 1,k + 1) \\ & \quad = \left( {\frac{Ds}{{2h^{2} }} + \frac{vs}{{4h}}} \right)C_{ij} (l - 1,k) + \left( {1 + FH_{i} - \frac{Ds}{{h^{2} }}} \right)C_{ij} (l,k) + \left( {\frac{Ds}{{2h^{2} }} - \frac{vs}{{4h}}} \right)C_{ij} (l - 1,k) \\ \end{aligned} $$

### Boundary numerate condition

With boundary condition is:

13 $$ C_{ij} (x,0) = C_{0ij} $$

14 $$ \frac{{\partial C_{ij} (x,t)}}{\partial x}{|}_{{x = l_{s} }} = 0 $$

15 $$ D_{i} \frac{{\partial C_{ij} (x,t)}}{\partial x}{|}_{{x = {0}}} = v_{j} [C_{ij} (0,t) - \overline{C}_{ij}^{\sec t} (t)] $$

$C_{0ij}$ represents the initial concentration distribution inside the columns at *t* = 0.In formula (14) and (15), $l_{s}$ and 0 represent respectively of end and initial position of column. In a simulated moving bed arrangement, the column inlet concentration $\overline{C}_{ij}^{\sec t} (t)$ depends on the section and the location of the column within the section as follow formula:

16 $$ \begin{aligned} & \overline{C}_{ij}^{{\text{I}}} (t) = \frac{{Q_{IV} C_{ij - 1} (l_{n - 1} ,t)}}{{Q_{I} }},\;Section\;I,\;1^{st} \;column \\ & \overline{C}_{ij}^{{{\text{III}}}} (t) = \frac{{Q_{II} C_{ij - 1} (l_{n - 1} ,t) + Q_{f} C_{fi} }}{{Q_{III} }},Section\;III,\;1^{st} \;column \\ & \overline{C}_{ij}^{\sec t} (t) = C_{ij - 1} (l_{n - 1} ,t),other \\ \end{aligned} $$

where $C_{f}$ the feed stream concentration and *Q* is the flow rate in each section, use formula (8–10), can get the boundary numerate condition:

17 $$ C_{ij} (w{ + 1},k){ + }C_{ij} (w{ + 1},k{ + 1}) = C_{ij} (w{ - 1},k + 1){ + }C_{ij} (w - {1},k) $$

18 $$ C_{ij} ({2},k{ + 1}){ + }C_{ij} ({2},k) - \frac{{4hv_{j} }}{{D_{i} }}\left( {C_{ij} (1,k) - \overline{C}_{ij}^{\sec t} (k)} \right) = C_{ij} ({0},k{ + 1}) + C_{ij} (0,k) $$

$w$ is the end of column. From formula (11), set $m = \frac{vs}{h},n = \frac{Ds}{{h^{2} }}$,substitute the Eq. (17) and (18) into formula (12), we get the next two boundary equation:

19 $$ \begin{aligned} & (1 + FH_{i} + 2n)C_{ij} (1,k + 1) - 2nC_{ij} (2,k + 1) \\ & \quad = \left( {1 + FH_{i} - 2n - \frac{8m(m + n)}{n}} \right)C_{ij} (1,k) + 2nC_{ij} (2,k) + \frac{{8m(m{ + }n)}}{n}\overline{C}_{ij}^{\sec t} (k) \\ \end{aligned} $$

20 $$ \begin{aligned} & - (m + n)C_{ij} (l - 1,k + 1) + (1 + FH_{i} + 2n)C_{ij} (l,k + 1) + (m - n)C_{ij} (l + 1,k + 1) \\ & \quad = (m + n)C_{ij} (l - 1,k) + (1 + FH_{i} - 2n)C_{ij} (l,k) - (m - n)C_{ij} (l + 1,k)\quad l \ne 1,w \\ \end{aligned} $$

21 $$ - 2nC_{ij} (n - 1,k + 1) + (1 + FH_{i} + 2n)C_{ij} (w,k + 1) = 2nC_{ij} (w - 1,k) + (1 + FH_{i} - 2n)C_{ij} (w,k) $$

denote the matric

$$ A = \left[ {\begin{array}{*{20}l} {1 + FH_{i} + 2n} \hfill & { - 2n} \hfill & 0 \hfill & \cdots \hfill & 0 \hfill \\ { - (m + n)} \hfill & {1 + FH_{i} + 2n} \hfill & {(m - n)} \hfill & \cdots \hfill & 0 \hfill \\ \vdots \hfill & \vdots \hfill & \vdots \hfill & \vdots \hfill & \vdots \hfill \\ 0 \hfill & \cdots \hfill & { - (m + n)} \hfill & {1 + FH_{i} + 2n} \hfill & {(m - n)} \hfill \\ 0 \hfill & \cdots \hfill & 0 \hfill & { - 2n} \hfill & {1 + FH_{i} + 2n} \hfill \\ \end{array} } \right] $$

$$ B = \left[ {\begin{array}{*{20}l} {1 + FH_{i} - 2n - \frac{8m(m + n)}{n}} \hfill & {2n} \hfill & 0 \hfill & {{\kern 1pt} \cdots } \hfill & 0 \hfill \\ {(m + n)} \hfill & {1 + FH_{i} - 2n} \hfill & { - (m - n)} \hfill & \cdots \hfill & 0 \hfill \\ \vdots \hfill & \vdots \hfill & \vdots \hfill & \vdots \hfill & \vdots \hfill \\ 0 \hfill & \cdots \hfill & {(m + n)} \hfill & {1 + FH_{i} - 2n} \hfill & { - (m - n)} \hfill \\ 0 \hfill & \cdots \hfill & 0 \hfill & {2n} \hfill & {1 + FH_{i} - 2n} \hfill \\ \end{array} } \right] $$

$$ w(k) = \left( {\begin{array}{*{20}c} {\frac{{m^{2} }}{m + n}\overline{C}_{ij}^{\sec t} (k)} & {0} & \cdots & {0} & {0} \\ \end{array} } \right)^{T} $$

So the Iterative equation is:

22 $$ AC_{ij} (k + 1) = BC_{ij} (k) + w(k) $$

For the discretization of linear isotherm, it can be proved that its discretization truncation error is $O(\Delta x^{2} ) + O(\Delta t^{2} )$. Similarly, for the linear isotherm, it can be proved that as long as the iterative step is small enough, its spectral radius is less than 1, it means $\rho (A^{ - 1} B) < 1$, that is, the iteration is stable.

## Simulation

### The initial parameter setting and simulation process

The simulated digitization system contains 8 packed columns which was 2-2-2-2 model, that is, each zone has two columns. Using finite difference method to solve the PDEs, setting the time step is 0.1 s, and the length of the string in space is divided into 100 parts. The initial parameters was shown in Table 2.

**Table 2:** The initial parameters for SMB.

Parameter	Value	Parameter	Value
*L* (cm)	25	*C*_*f,i*_ (g L^−1^)	5
*d* (cm)	0.46	θ (min)	3
*H*_*A*_	0.0001	*Q*_*I*_ (cm^3^ min^−1^)	7.080
*H*_*B*_	0.45	*Q*_*II*_ (cm^3^ min^−1^)	4.607
*D*_*A*_ (cm^2^ min^−1^)	0.3	*Q*_*III*_ (cm^3^ min^−1^)	6.716
*D*_*B*_ (cm^2^ min^−1^)	1.255	*Q*_*IV*_ (cm^3^ min^−1^)	4.519
ε	0.8		

**Figure 3:**
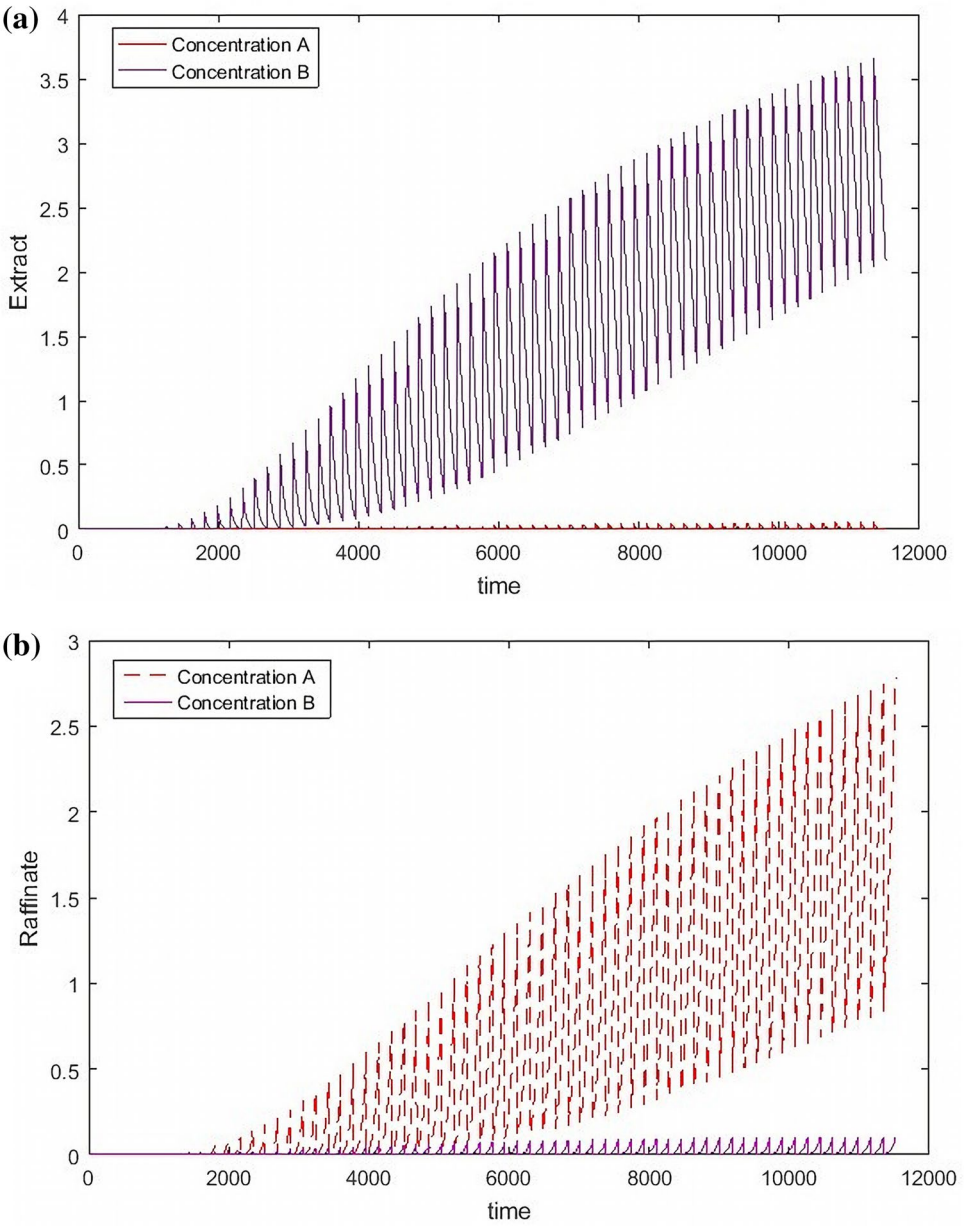
The concentration separation results of outlets.

All the digitization calculations were conducted in MATLAB (Version R2016a 9.0.0.341360, 64bit Win 64, and available from https://www.mathworks.com) on a PC equipped with Intel core i7-3770 K 3.53 GHz and 16 GB RAM running Windows 10. The simulation result show in Fig. 3.

From the Fig. 3, the results show that the forward difference method is used to digitize the SMB process; the data graph obtained is similar to that simulated by traditional SMB software. Now SMB simulation software is based on finite method, which shows that the digitalization process is effective, and the digitalization result is controllable for the intermediate process, which is different from the traditional finite difference method. For the SMB software mentioned in this paper, it is just to explain that different separated substances use different software in the market, there is no unified available software, and their software is not used in this paper.

### Sensitivity analysis of the parameters of the SMB process

The following figure shows that changes in SMB parameters affect the final equilibrium concentration, and were drawled in MATLAB (Version R2016a 9.0.0.341360, 64bit Win 64, and available from https://www.mathworks.com). Now we consider the parameter how to affect the system’s concentration and sensibility.

**Figure 4:**
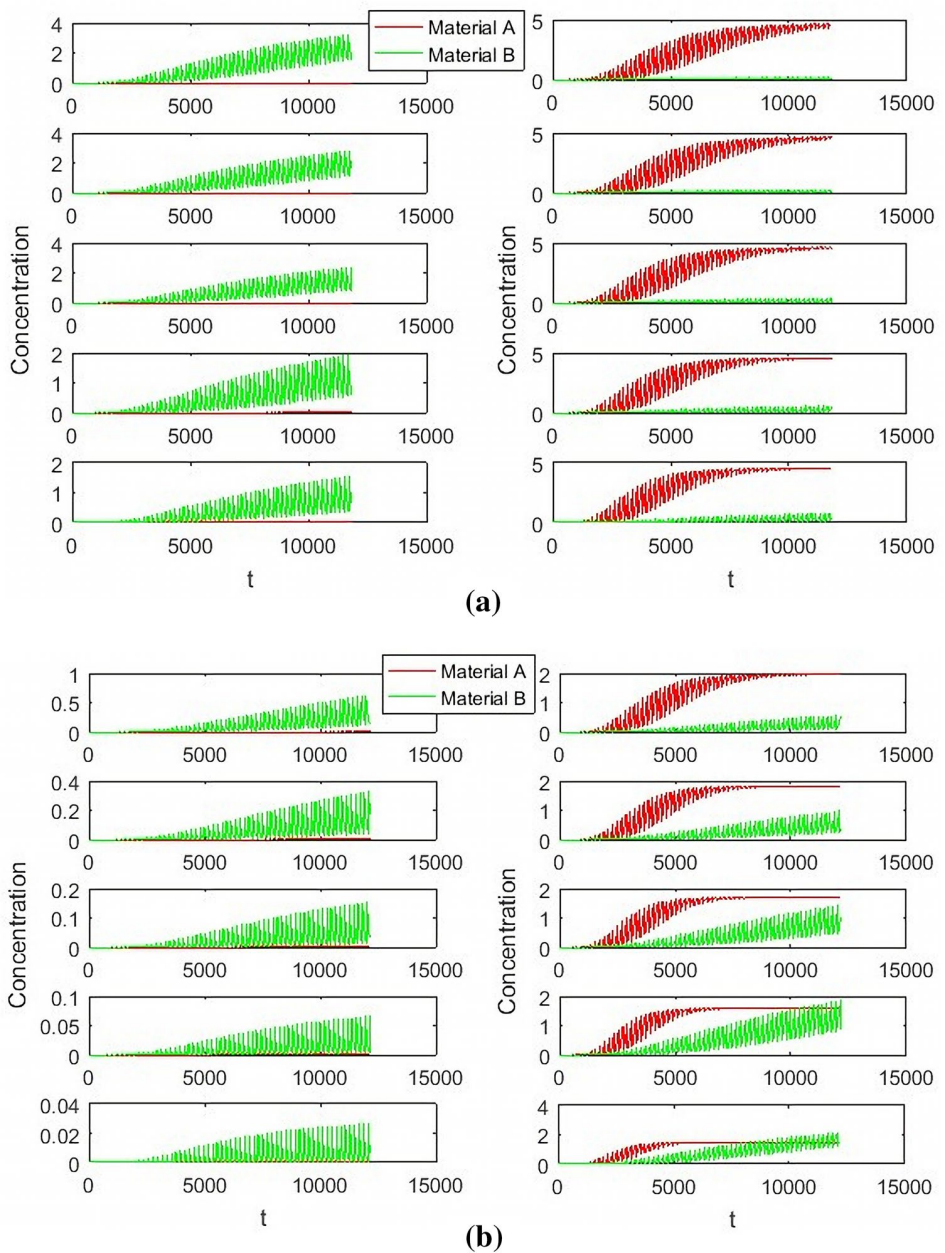
The influence of switching time on SMB process.

In the Fig. 4, switch time change from 186 to 195. Then the switching time has severe effect on SMB process, it affects not only the success of the final separation process, but also affects the final concentration of separation and the time when the separation reaches steady state. When the switching time is short, two substances could not be successfully separated, the separation concentration is not large enough, but it can reach steady state time quickly. With the increase of switching time, the separation gradually succeeds, and the concentration of separation is large enough, but the steady-state time to be achieved also increases.

**Figure 5:**
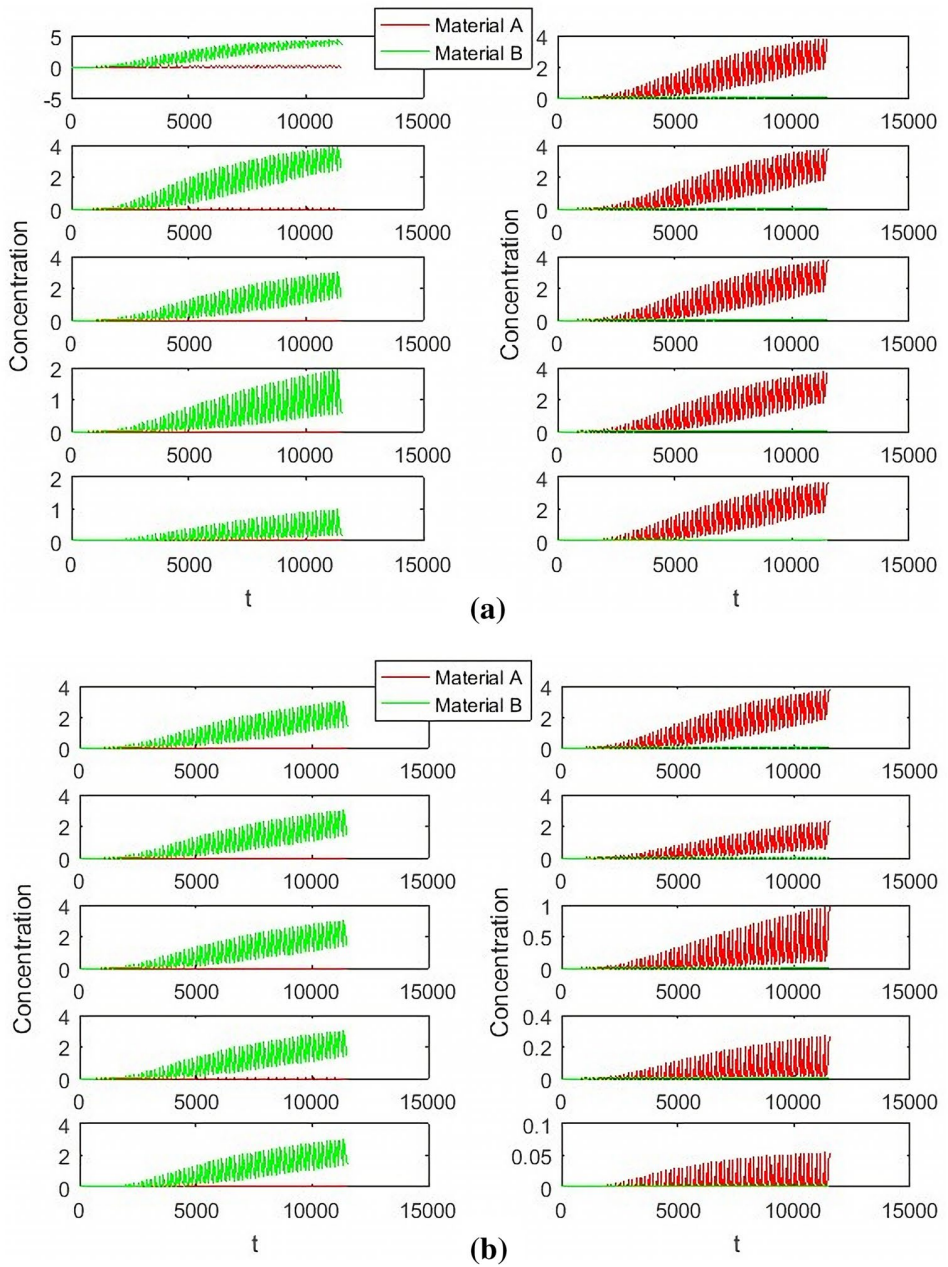
The influence of Zone 1,2 Flow rate for concentration.

In the Fig. 5, *Q*_*I*_ change from 6.8 to 7.7, when the concentration of extract about material B is decreases with the increase of the zone1 flow rate, but The concentration of raffinate about material A is almost unchanged. *Q*_*II*_ change from 6.36 to 6.76, when *Q*_*I*_ = 6.8, *Q*_*III*_ = 8.4, *Q*_*IV*_ = 2. The concentration of extract about material B is almost unchanged ,but material A is increases with the increase of zone2 flow rate.

**Figure 6:**
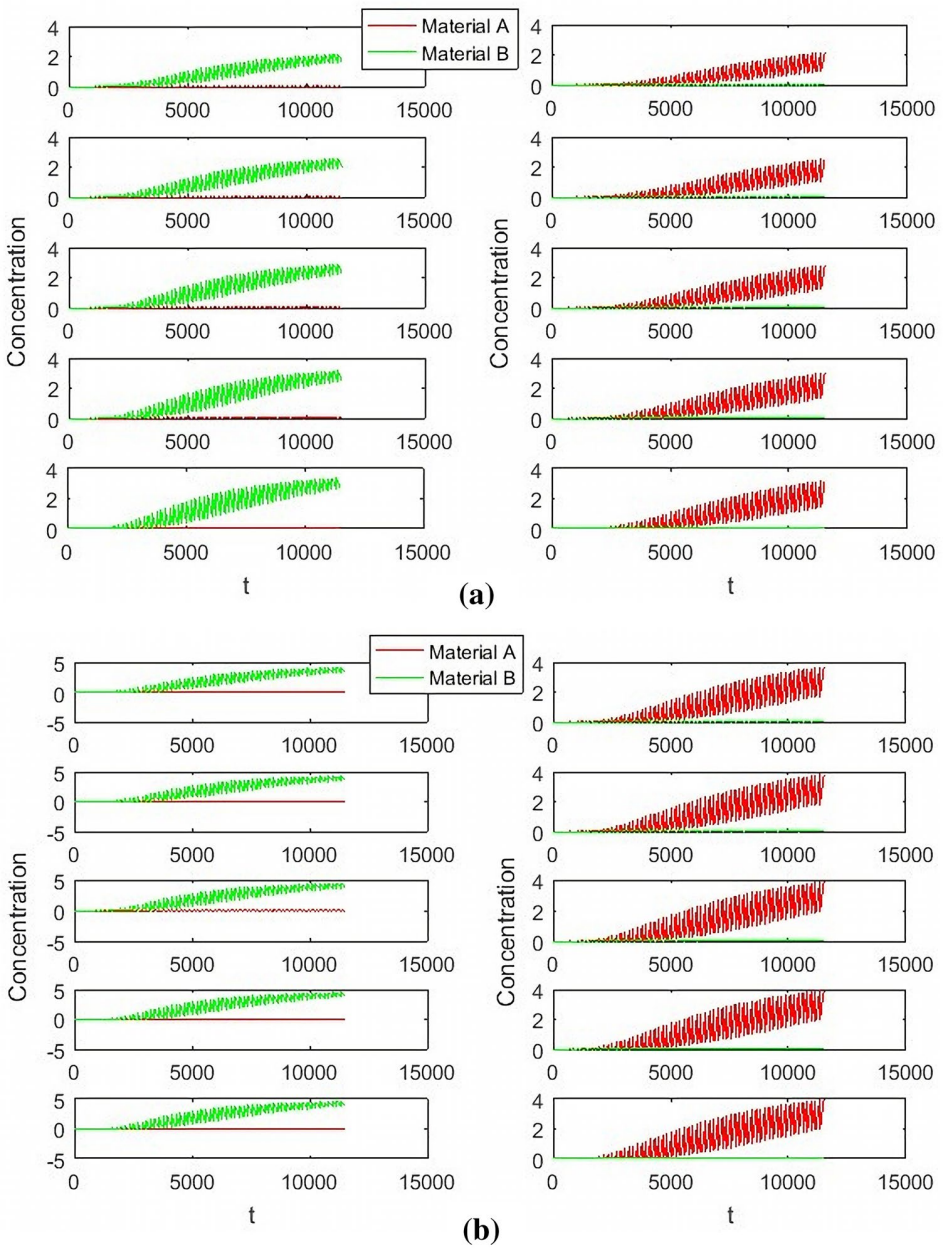
The influence of Zone 3 Flow rate for concentration.

In the Fig. 6 of left, *Q*_*II*_ change from 7 to 7.9, when *Q*_*I*_ = 6.8, *Q*_*II*_ = 6.7, *Q*_*IV*_ = 2. The concentration of extract about material B is decreases with the increase of the zone3 flow rate, and the concentration of raffinate about material A is also increased. In the Fig. 6 of right, *Q*_*III*_ change from 8 to 8.8, when *Q*_*I*_ = 6.8, *Q*_*II*_ = 6.7, *Q*_*IV*_ = 2. The concentration of extract about material B is slight increases with the increase of the Zone3 flow rate, and the concentration of raffinate about material A is also slight increased.

**Figure 7:**
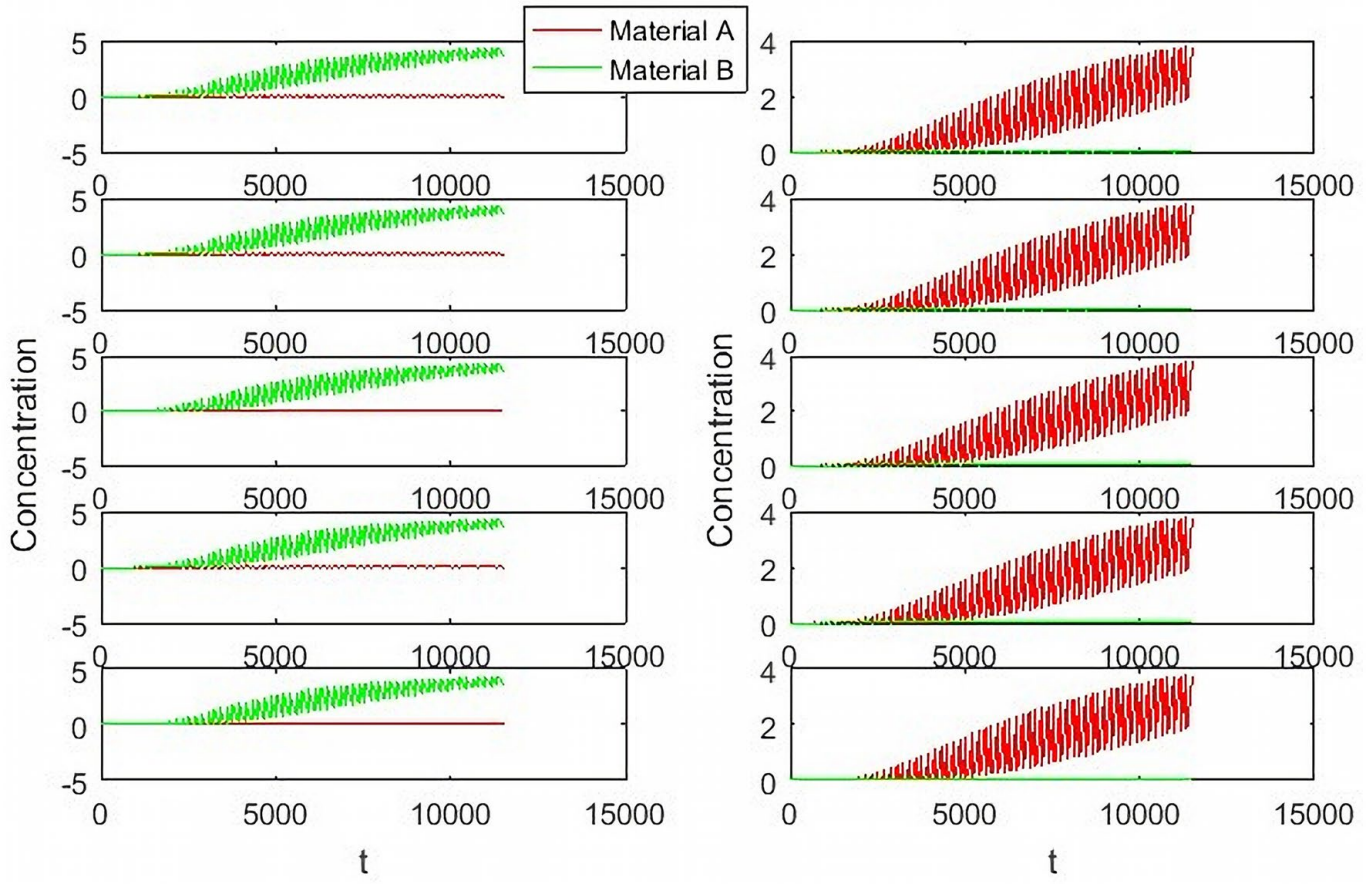
The influence of Zone 4 Flow rate for concentration.

In the Fig. 7, *Q*_*IV*_ change from 2 to 4, when *Q*_*I*_ = 6.8, *Q*_*II*_ = 6.7, *Q*_*III*_ = 8.4. The concentration of extract about material B is almost unchanged, and the concentration of raffinate about material A is also almost unchanged.

## Hierarchical Fuzzy controls

### Fuzzy rule and controller structure

In the hierarchical fuzzy control, the input variables contain error, first-order error difference, the formula is as follows:

23 $$ e_{1} = desired \, B - C_{E,B} $$

24 $$ e_{2} = desired \, A - C_{R,A} $$

25 $$ \Delta e_{1} = e_{1} (k) - e_{1} (k - 1) $$

26 $$ \Delta e_{2} = e_{2} (k) - e_{2} (k - 1) $$

$C_{E,B}$ represents extract material *B* of concentration, and $C_{R,A}$ represents raffinate material *A* of concentration. Desired B represents the control goal of concentration of B at the extract port, and Desired A represents the control goal of concentration of A at the raffinate port.

**Figure 8:**
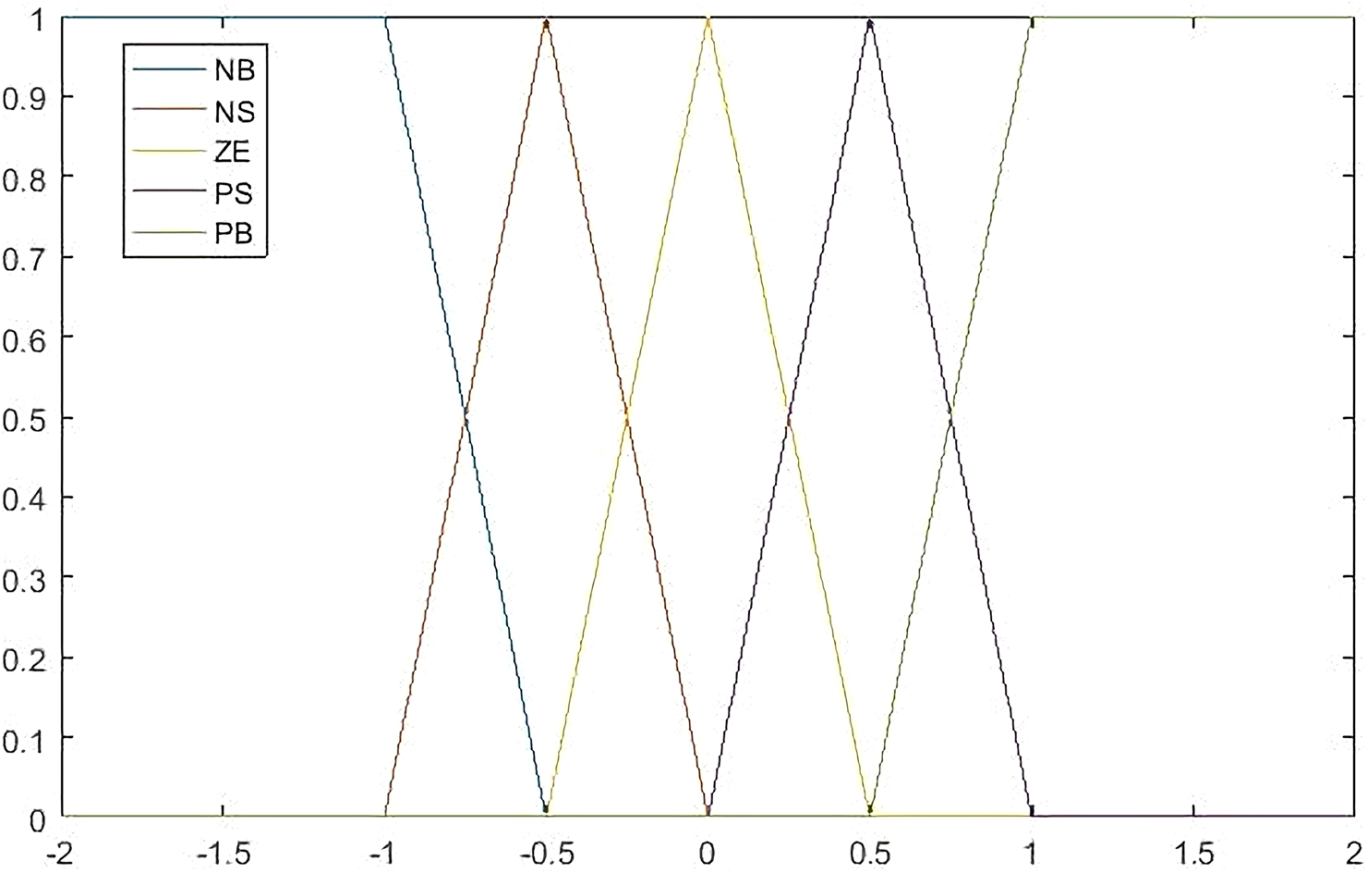
Membership function.

Next, the input variables need to be fuzzier. The fuzzy system defines five linguistic variable values on errors: *NB, NS, ZE, PS, PB* and five linguistic for the first order difference of errors: *NB, NS, ZE, PS, PB.* All membership functions are triangular function, because it belongs to linearity, unlike the long tail of Gauss function, the membership function shown in Fig. 8.

Then defines the output control parameter flow rate $\Delta {\text{Q}}_{{\text{I}}} ,\Delta {\text{Q}}_{{{\text{II}}}} ,\Delta {\text{Q}}_{{{\text{III}}}}$ during the SMB operation process, three independent fuzzy controllers act on three regions, for the input of the third region, we use the weighted average of the input errors of the first region and the second region, taking the single pole fuzzification value where the center value is (*NB,NS,ZE,PS,PB*) = {0.15, 0.1, 0, − 0.1, − 0.15} for $\Delta {\text{Q}}_{{\text{I}}}$ (*NB,NS,ZE,PS,PB*) = {0.006, 0.004, 0, − 0.004, − 0.006} for $\Delta {\text{Q}}_{{{\text{II}}}}$. (*NB, NS, ZE, PS, PB*) = {0.08, 0.05, 0, − 0.05, − 0.08} for $\Delta {\text{Q}}_{{{\text{III}}}}$.

Next, we draw on the principle of kinematics to formulate the corresponding fuzzy rule as Table 3. For the force direction of $\Delta {\text{Q}}$, according to the sensitivity analysis of regional velocity, the increase of regional velocity plays the role of negative force, that is, pulling force, and the decrease of regional velocity plays the role of pushing force. Of course, these analyses are effective in a local analysis interval.

**Table 3:** Fuzzy rule for $\Delta Q_{i}$.

$\dot{e}$	*e*				
	*NB*	*NS*	*ZE*	*PS*	*PB*
*NB*	*NB*	*NB*	*NB*	*NS*	*PB*
*NS*	*NB*	*NS*	*NS*	*ZE*	*PB*
*ZE*	*NB*	*NS*	*ZE*	*PS*	*PB*
*PS*	*NB*	*ZE*	*PS*	*PS*	*PB*
*PB*	*NB*	*PS*	*PB*	*PB*	*PB*

Mamdani operator, basic complementary operation and Mamdani fuzzy reasoning rule are used in the preceding part of reasoning^37^.

The last step is to defuzzifier, here, we choose the central weighted average method to defuzzifier. From the fuzzy rule base and the membership function of the input variables, we can see that each input will start four rules, and the strength of these four rules, that is, the weight is calculated by the T-norm of the if part. The formulas are shown in (27)–(29).

27 $$ V = T(u_{1} ,u_{3} ) \times y_{1} + T(u_{1} ,u_{{4}} ) \times y_{2} { + }T(u_{2} ,u_{3} ) \times y_{3} + T(u_{2} ,u_{{4}} ) \times y_{4} $$

28 $$ W = T(u_{1} ,u_{3} ) + T(u_{1} ,u_{{4}} ){ + }T(u_{2} ,u_{3} ) + T(u_{2} ,u_{{4}} ) $$

29 $$ U = \frac{V}{W} $$

$u_{1} ,u_{2}$ are the error values after fuzzification, $u_{{3}} ,u_{{4}}$ are the first-order differences after fuzzification, and $y_{1} \ldots y_{{4}}$ corresponds to the unipolar values of the four starting rules, that is the unipolar values of the Then part. T is the product type T-norm, and the final output is $U$.

**Figure 9:**
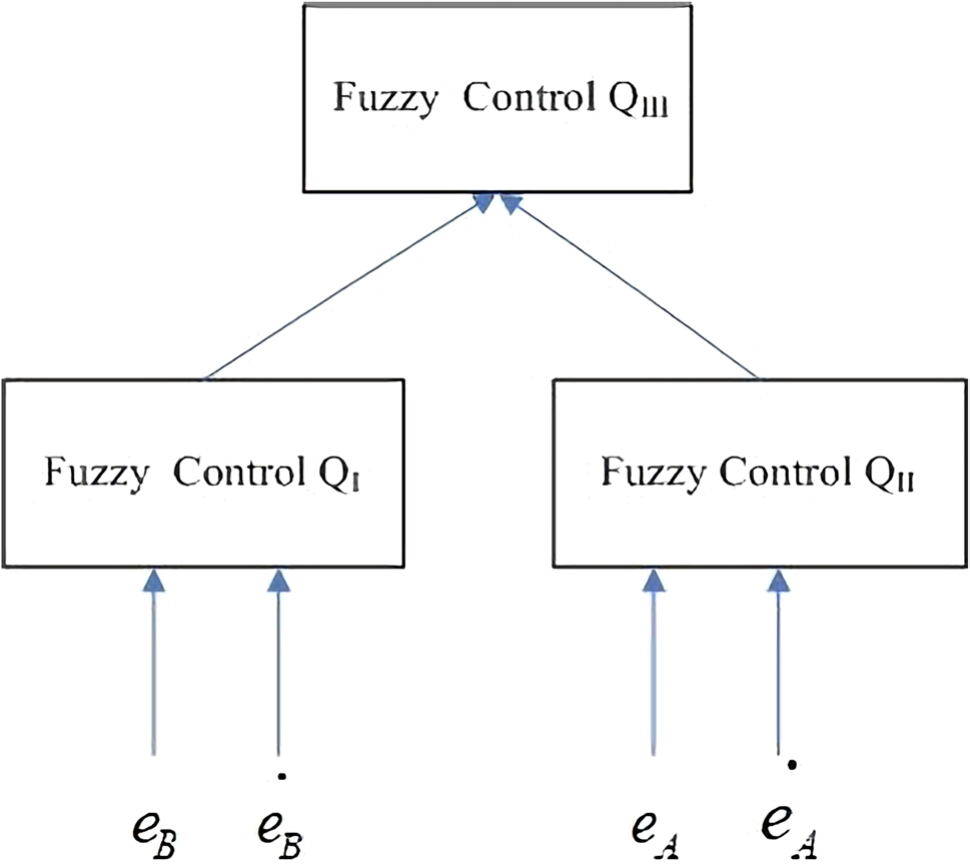
Hierarchical Fuzzy control.

The control structure is shown in Fig. 9. Then we use the controller to control the concentration of material.

In the control process, we need to set an observer. If the output force according to the error input of each order violates the restriction of formula (7), the force adjustment will be cancelled.

### Concentration control result

The following figures show the control results of extract and raffinate port.

**Figure 10:**
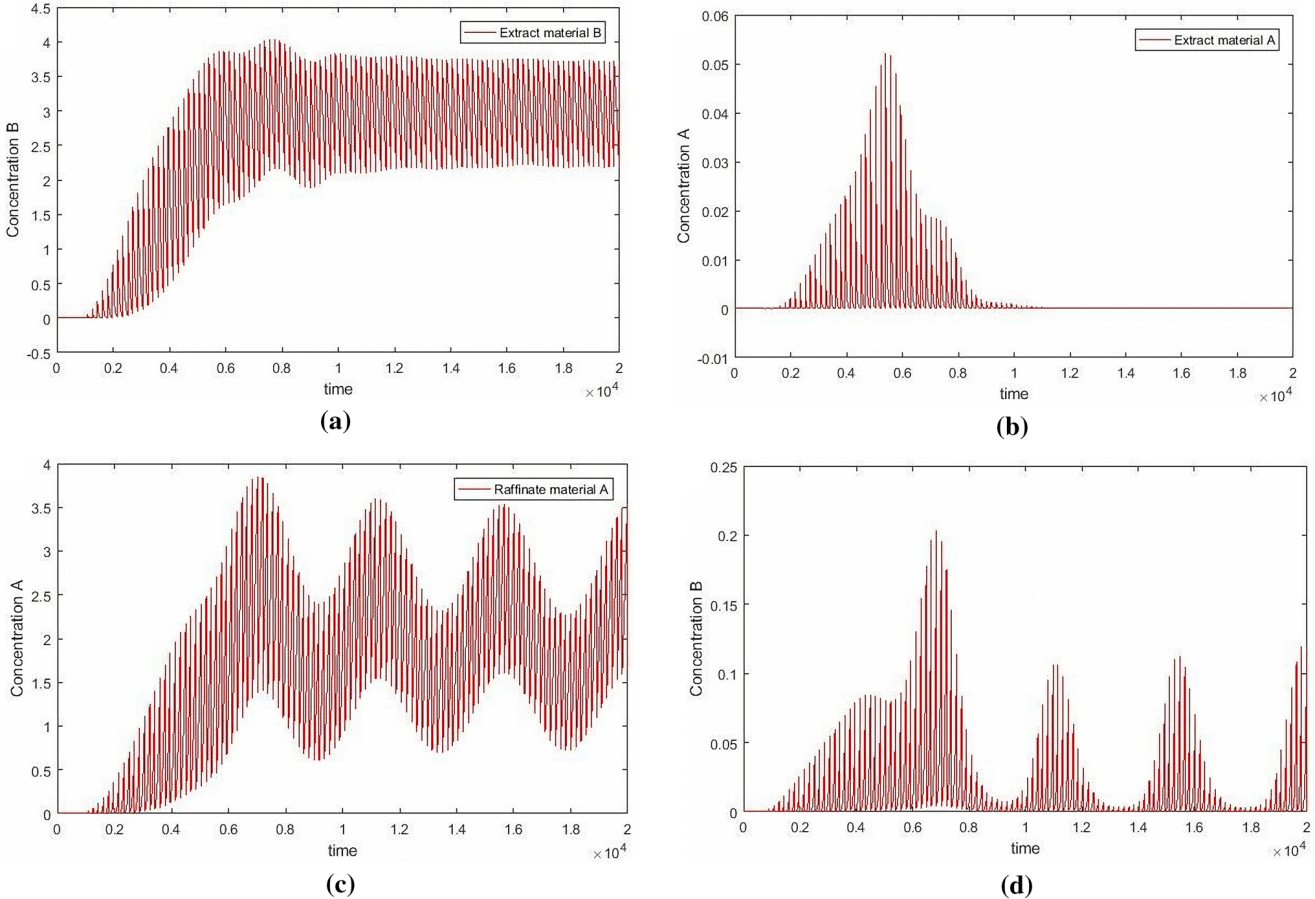
Hierarchical Fuzzy Rule Control for concentration, desired B = 3, desired A = 2, Switch time = 180 s.

As can be seen from Fig. 10, the two materials oscillate back and forth periodically in the center of the target concentration, because the switch time itself is outside the control system. Even if it is included in the system, oscillation cannot be eliminated, because the concentration will change periodically when the SMB reaches a stable state.

In order to better understand the change trend, the data are processed by periodic average:

30 $$ \mathop C\limits^{\_}_{E,B,t} = \frac{{\int_{t - T}^{t} {C_{E,B,t} dt} }}{T} $$

31 $$ \mathop C\limits^{\_}_{R,A,t} = \frac{{\int_{t - T}^{t} {C_{R,A,t} dt} }}{T} $$

$\overline{C}_{E,B,t}$ is the concentration of material B at the extract port at time *t*, $\mathop C\limits^{\_}_{R,A,t}$ is the concentration of material A at the raffinate port at time *t*, the simulation result is shown below.

**Figure 11:**
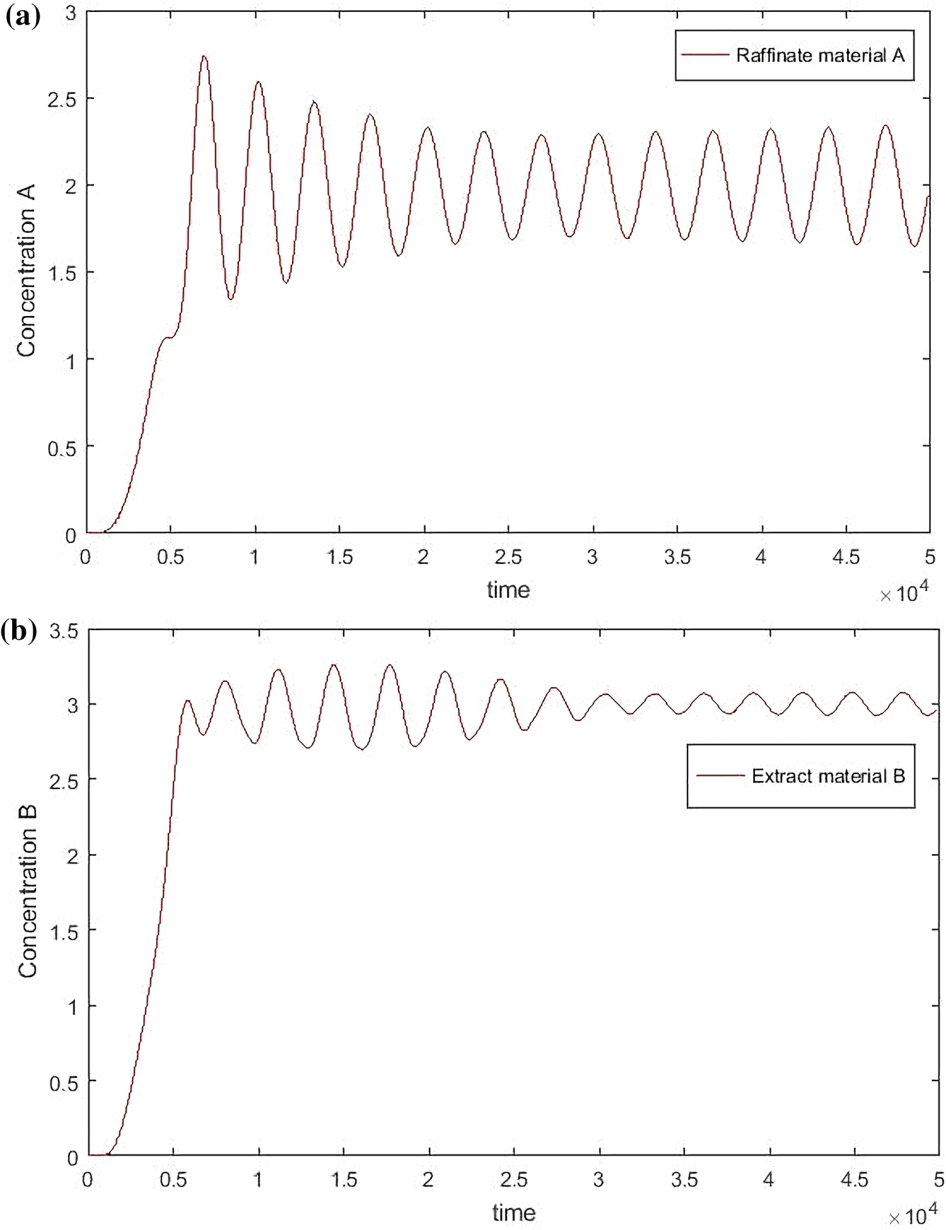
Hierarchical Fuzzy Rule Control for concentration, desired *B* = 3, desired *A* = 2.

**Figure 12:**
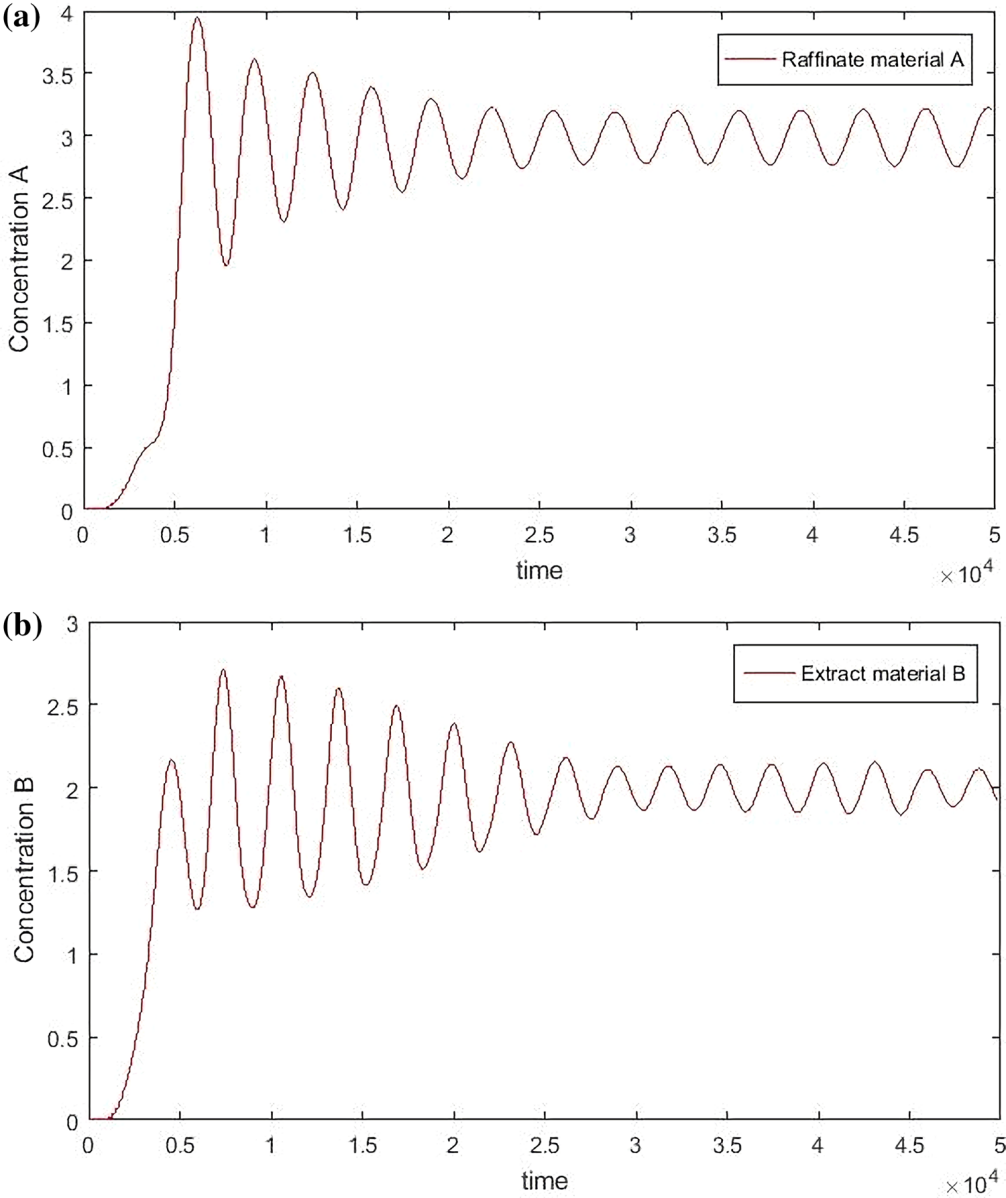
Hierarchical Fuzzy Rule Control for concentration, desired *B* = 2, desired *A* = 3 Switch time = 180 s.

It can be seen from Figs. 11 and 12 that the hierarchical fuzzy control can achieve good effect under different concentrations and switching times, but it cannot remove the fluctuation near the target value. Because the change of material concentration will re-enter a cycle every time, and volatility cannot be eliminated within the system. However, it is necessary to compare the hierarchical fuzzy controller with other controllers to determine whether the fuzzy controller has better performance.

### Controller comparison

**Figure 13:**
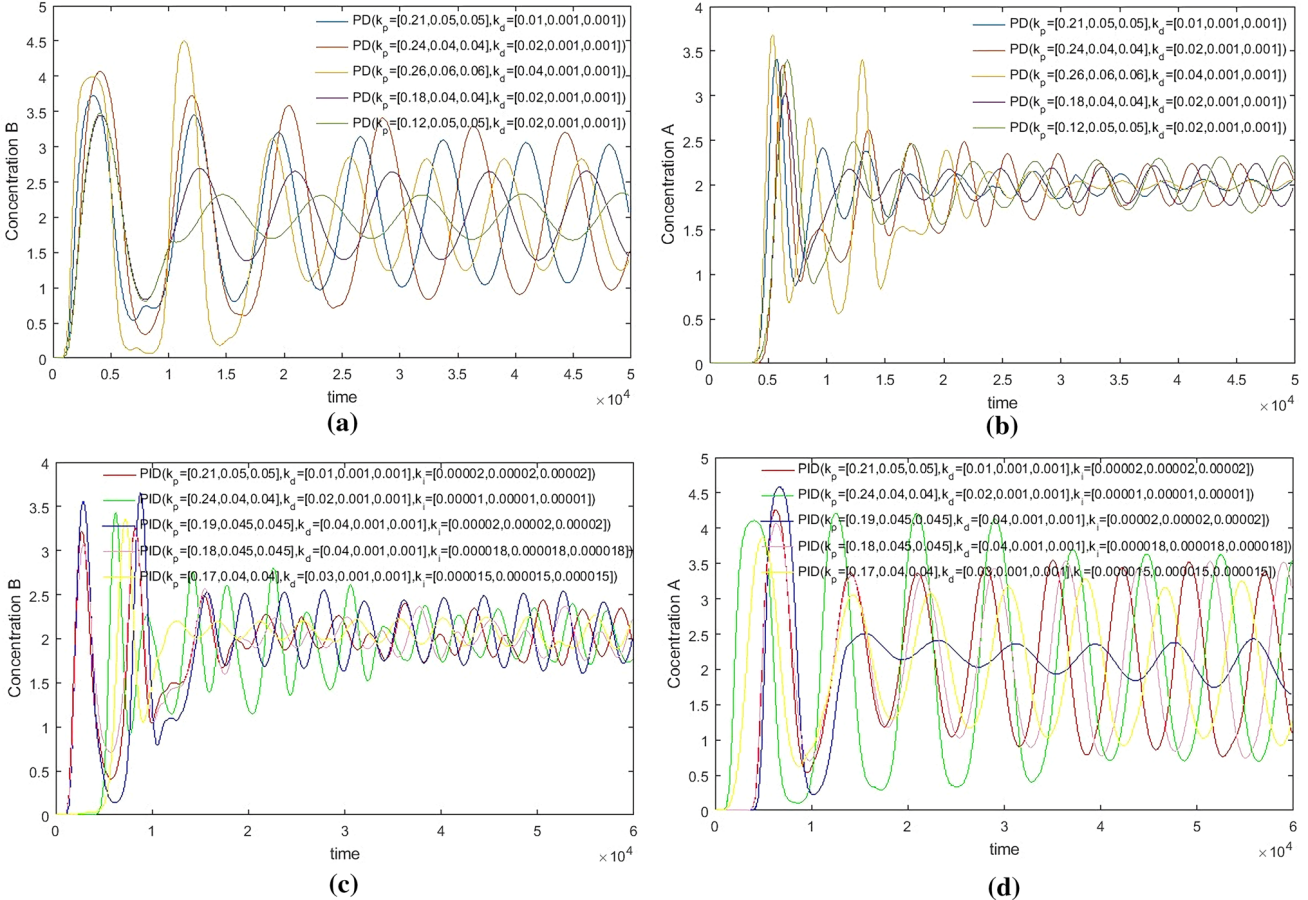
PD and PID Controller under different parameters, Switch time = 180 s.

In this part, we compare the hierarchical fuzzy controller with PD and PID controller, the results shown in the follow figures:

From Fig. 13, it can be seen that the parameter adjustment of PD and PID controllers can’t achieve the perfect control of two outlets at the same time. Either the E outlet effect is good or the R outlet effect is good. When compared with the hierarchical fuzzy controller, the study chooses two parameters with moderate effect as comparison.

**Figure 14:**
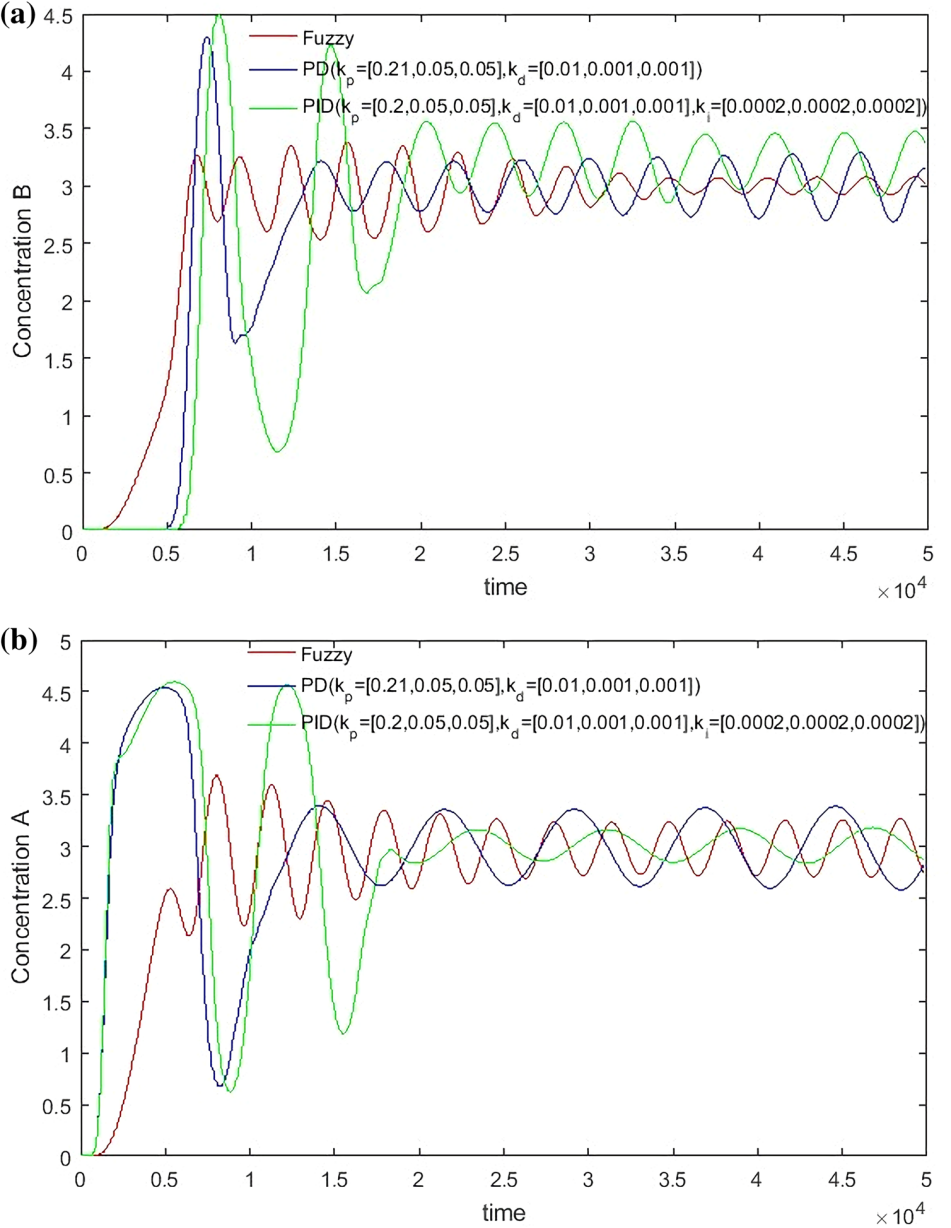
Desired B = 3, desired A = 3, Switch time = 180 s.

**Figure 15:**
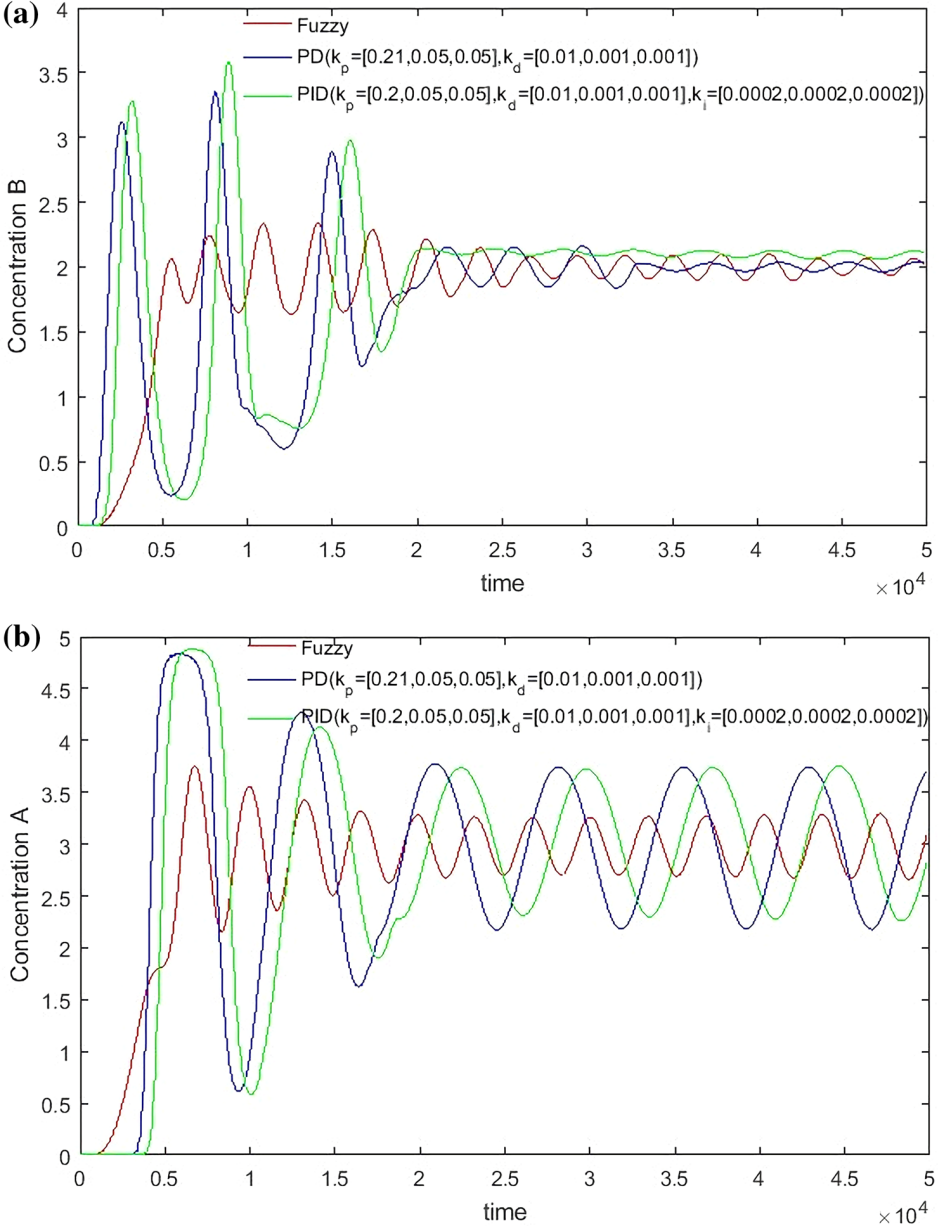
Desired B = 2, desired A = 3, Switch time = 180 s.

**Figure 16:**
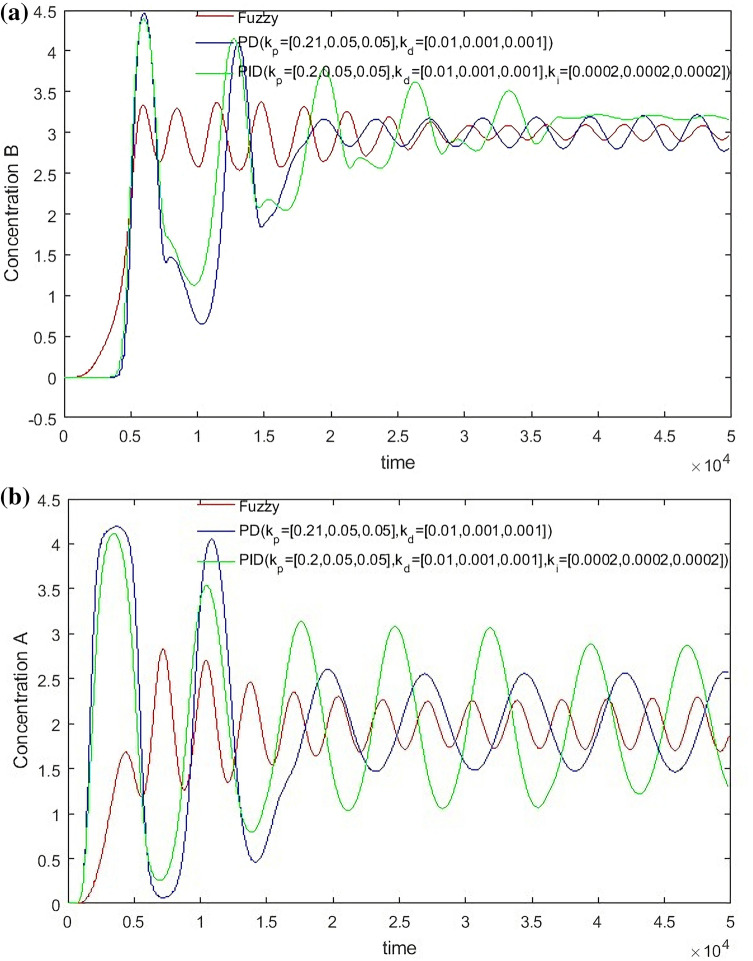
Desired B = 3, desired A = 2, Switch time = 180 s.

**Figure 17:**
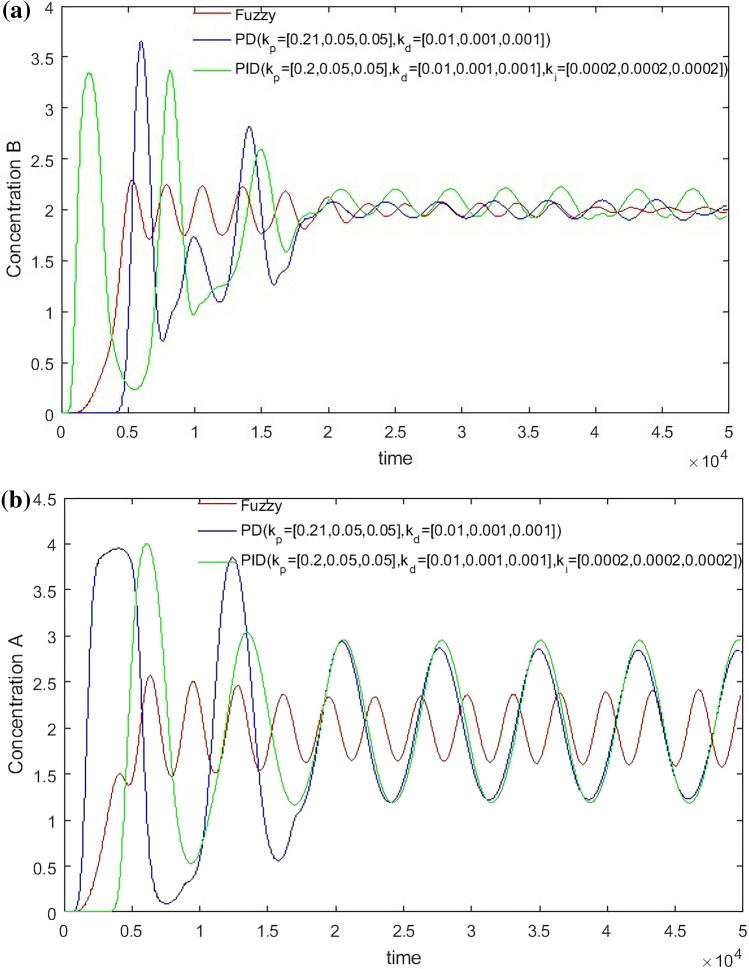
Desired B = 2, desired A = 2, Switch time = 180 s.

From Figs. 14, 15, 16 and 17, it can be seen that in some cases, PD or PID may be better, but overall, PD and PID can’t adapt to the control of different concentrations, in practice, it is impossible to control a concentration to readjust a parameter, and PD and PID can’t achieve very good control of the concentration of both substances. On the contrary, the performance of the hierarchical fuzzy controller is very stable. Regardless of the concentration of the control, the control of both substances achieves good results at the same time, and the time for the hierarchical fuzzy controller to reach steady state is a little shorter.

**Figure 18:**
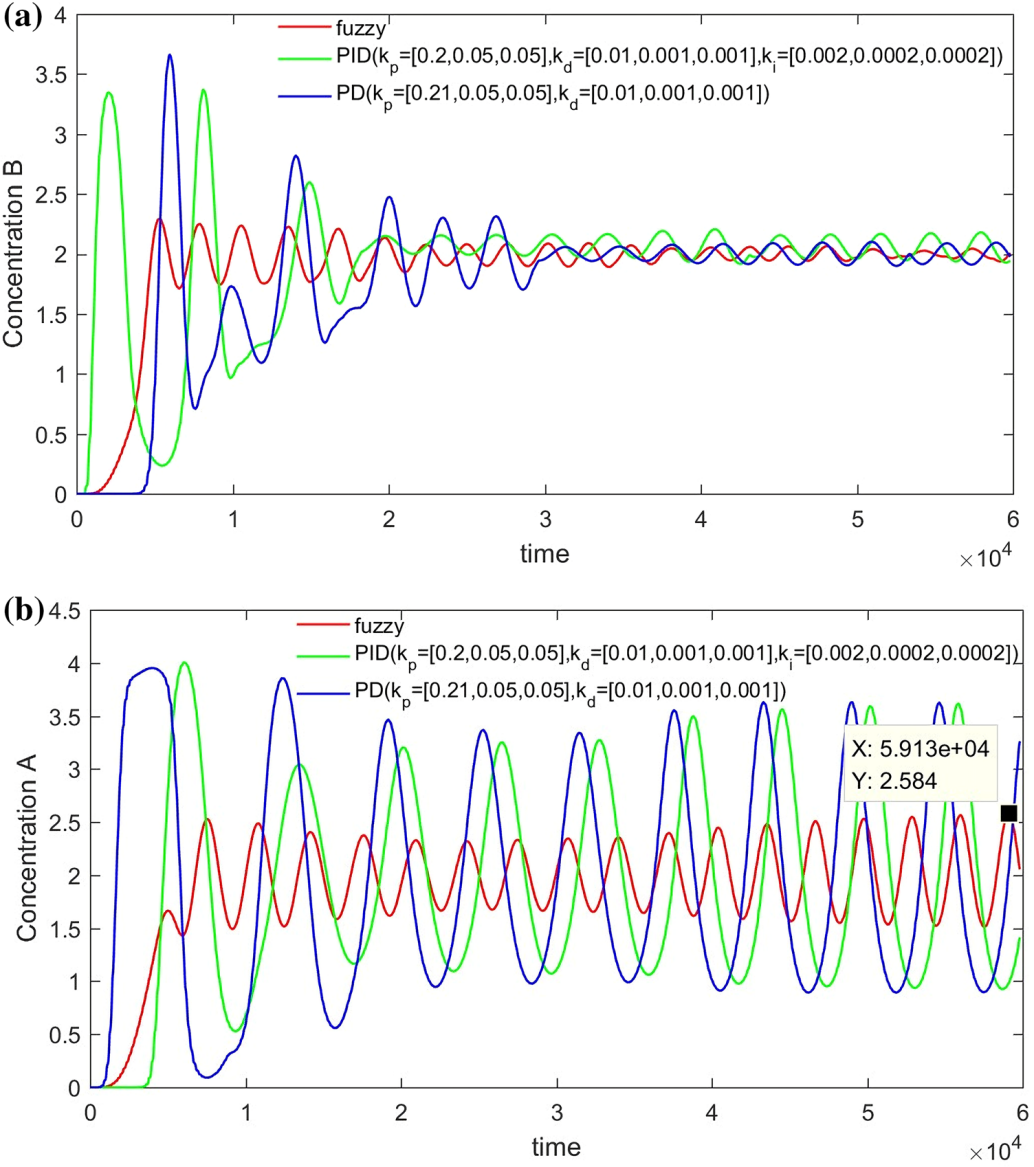
Under the change of feed port concentration $C_{{_{f} }} = 5 \to 10$.

As can be seen from Fig. 18, the change of feed inlet concentration has no impact on the concentration control of extract outlet material B, but the concentration fluctuation of raffinate outlet material A becomes larger. Compared with PD and PID controllers, the fluctuation of hierarchical fuzzy controller is smaller and more robust.

**Figure 19:**
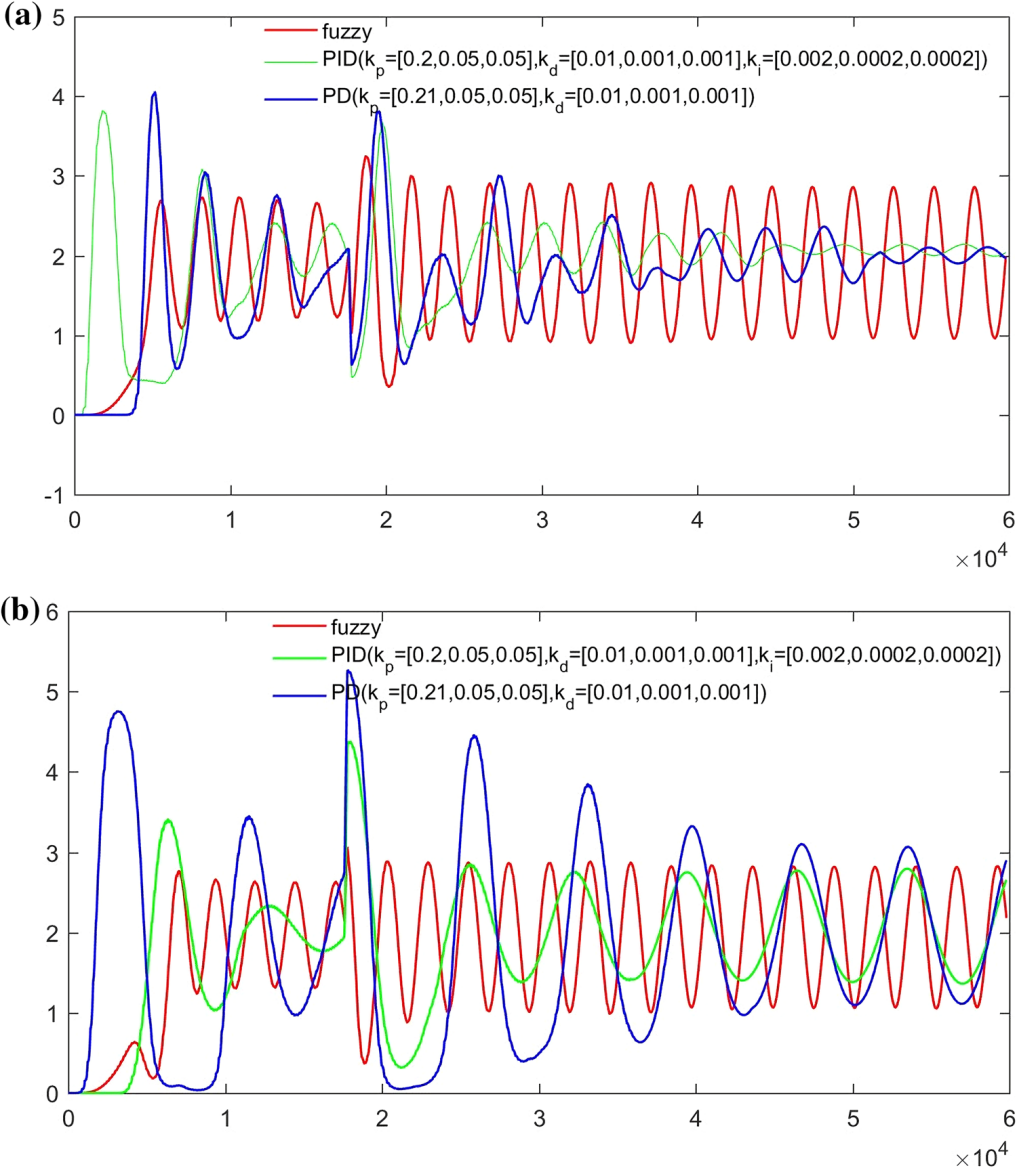
Under the change of switch time $\theta = 178s \to 182s$.

It can be seen from Fig. 19 that the change of switching time has a great impact on the hierarchical fuzzy controller, PD and PID controller. Although the fuzzy controller enters a stable state faster, the oscillation amplitude becomes large, which has no advantage over Pd and PID controllers. For the parameter of switching time, the controller needs to be further improved to obtain better performance.

## Conclusion

In this paper, the SMB process is digitalized by using centered difference method, in this way, the concentration data of materials changing with time and space can be generated, and the data trend charts with different parameters can be obtained. Through observation, it is found that different parameters do affect the concentration of separation, which brings great value to practical application. Using hierarchical fuzzy controller, research found that in the case of successful separation, controlling the concentration of material can achieve good results, but the switching time is outside the control system, it can’t fundamentally eliminate the fluctuation of concentration around the target value, no matter how to adjust the switching time can’t be achieved, because the existence of switching time is to ensure the material content condition for separation. Finally, through the comparison of three kinds of controllers, it is found that the hierarchical fuzzy controller can control good without knowing the system parameters too accurately and full understanding of the mechanism model of the system. In addition, the performance of the fuzzy controller is more stable when the feed inlet concentration changes, but for the change of switching time, the fuzzy controller has no advantage, and there is a disadvantage of using fuzzy controller in SMB system; we need to find a monotone interval of the change of control parameters to the change of target solubility. Unfortunately, the control region used in the study is a local region, and the control analysis of control variables in the global feasible region can’t be realized. The future research direction is to use artificial intelligence reinforcement learning to predict the global feasible region.
